# Monthly Summary

**Published:** 1882-05

**Authors:** 


### MONTHLY SUMMARY.

*Anatomical Anomalies.*—Dr. J. G. Wiltshire, of Baltimore, mentions, in the *Maryland Medical Journal*, the occurrence, in a colored subject of fine physique, of a peculiar series of anomalies in the position of the heart and the arrangement of some of the arteries. The heart was found on the right side, with its base at the proper level, corresponding with the upper borders of the third costal cartilages, with its apex pointing to the right, resting on the diaphragm at a space between the fifth and sixth ribs. It was normal in size and appearance, and had apparently taken this position in its cavity without any agent to determine the misplacement.

From the left side of the heart the arch of the aorta came off, and in every way behaved as it ought to have done, save in giving off its branches. The arteria innominata was wanting, its branches coming off from the arch in the following order:

The right common carotid arose from the upper face of the tranverse part of the arch, where it begins to descend toward the third dorsal vertebra, and then it ascended, in front of the trachea, to the right side of the neck, to its usual point of bifurcation into the external and internal carotids; the former gave off no branches until it reached a point opposite the symphsis of the chin, where a short axis was thrown off, from which the facial, lingual and superior thyroid arteries arose. The latter (internal carotid) presented nothing that was at all irregular in its behavior.

The left common carotid artery was derived from the outer face of the descending part of the arch and ascended, upon its old bed, to its usual point of bifurcation, acting as did its fellow on the opposite side.

The left subclavian arose from the decending part of the arch of the aorta, at a point where it crosses, and passed upward and outward to its usual line of passage to the anterior border of the first rib.

Nothing more occurred to the arterial tree to interest us, until it passed into the abdomen, where the cœliac axis came off from the posterior face of the aorta, and passed to the right of the latter to its front, where the hepatic, gastric and splenic arteries were given off. There were two renal arteries on the right side, whereas there was only one on the left.—*Medical and Surgical Reporter.*

---

*Large Odontome Removed from the Lower Jaw.*—At a recent meeting of the Pathological Society, Mr. Christopher Heath read notes of a case in which he had removed a large odontome from the lower jaw. This was one of the rare tumors described by Broca as "odontomes odontoplastiques," and consisted of a mass of dentine studded with nodules of enamel. The mass weighed 315 grs, and measured 1½ in. by 1¼ in. The patient was a young lady of eighteen, who had never been able to close her teeth properly, but otherwise was supposed to have gone through the first and second dentitions naturally. Last Christmas she had pain and uneasiness about the right angle of the lower jaw; and in April her father, a dental surgeon, extracted the second bicuspid tooth, there being no molars then present. A dentist, who was frequently consulted, thought he detected an encysted tooth, and tried to extract it with the elevator. The result was an acute attack of periostitis. Profuse suppuration ensued, and on firm pressure near the angle pus could be forced up from the interior of the bone. Under treatment the inflammation subsided, and the patient went to the seaside, and on her return there was apparently some exposed bone, with greatly hypertrophied mucous membrane on each side. A month later, after imprudent bathing, sudden increase of pain and swelling took place, and she consulted Mr. Heath, who found great enlargement of the bone, with a fungus-like growth in the mouth, and apparently bare bone, the appearances closely resembling those ordinarily found in a case of sarcoma of the jaw. An operation involving removal of a portion of the jaw was declined, and the swelling slowly diminished again. In September Mr. Heath undertook an operation for removal of the supposed sequestrum of bone, and after considerable trouble succeeded in elevating the mass described from its bed, since which the jaw has slowly contracted to its proper stage.—*London Lancet.*

*A New Cure for Alveolar Abscess.*—The perfect application of any medicament to all parts of a large and inaccessible abscess always presented considerably mechanical difficulties, and it has occurred to me that, for the hydraulic or pneumatic pressures which are usually applied, there might be substituted some rapid chemical evolution within the cavity. I carried out this idea of injecting into a large abscess in my own mouth which had resisted all the ordinary applications of carbolic acid, creosote, and every other known remedy, as strong a solution as I could obtain of peroxide of hydrogen. This, if cold and rapidly injected, almost immediately afforded a rapid evolution of oxygen upon the whole surface of the abscess, and a more satisfactory antiseptic than nascent oxygen could scarcely be conceived. The liquid when injected was perfectly clear, but the operation was immediately followed by an enlargement of the cavity and the exudation of a white milky froth. The result was extremely satisfactory, a single injection of the peroxide effecting a complete cure.—*Dr. Walter Coffin.*

---

*Communicability of Syphilis by Suckling.*—In the *Giornael Italiano di Soresina* (1880, p. 15,) Professor Scarenzio relates a case, which, if free from error in observation; is of much importance. A healthy young woman, aged 19, married, in December, 1874, a vigorous young man, lately returned from his regiment. In September, 1875, she gave birth to a weakly infant, which developed symptoms characteristic of inherited syphilis. When seven months old, its lips became the seat of erosions and ulcerations at the commissures. At the same time the mother, who had exclusively suckled the child, saw a chancre, accompanied with enlarged glands, develop on the right nipple, shortly followed by a characteristic syphilitic eruption. She gave birth to another syphilitic child in July, 1877. The author infers, from this case, that a woman may bear a syphilitic child to a syphilitic father, and remain herself uninfected; and, furtner, that it is imprudent to cause a syphilitic infant to be nursed by its mother, unless the latter should previously have exhibited symptoms of syphilis.—*Medical and Surgical Reporter.*

---

*Death from Carbolic Acid.*—Rev. James Cameron, of Oakland, lost his life through swallowing a draught containing carbolic acid. The mixture was designed for external application and the bottle containing it stood by a larger bottle containing a tonic which it was the intention to give him. He took the poisonous dose from a wine glass, and after swallowing half of it remarked that it was very strong, and then drank the remainder. There is something curious in the published account, which states that the mistake was discovered immediately, and an attempt made to give an emetic, but that he could not swallow; that the attendants forced down as much magnesia and oil as they could, but he "died in a few minutes." How a small draught so dilute as to admit of his voluntarily swallowing the second portion after tasting the first, could have produced death in so short a time is not comprehensible.—*Pacific Medical and Surgical Journal.*

---

*Dentistry as a Specialty.*—At a recent banquet of the Chicago Dental Society some interesting remarks concerning the present and future of dental surgery were tendered W. W. Allport, M. D., D D. S. After alluding to the early efforts of Dr. Harris and others to establish a chair of oral surgery in some of our medical colleges, and the final establishment of our first dental college in Baltimore, the speaker called atttention to the present facilities for dental education in this country. Dr. Allport holds that a general medical education is as necessary as a foundation in dentistry as in ophthalmology, gynecology, or any other department of operative medical science, and thinks that at no distant day such a foundation will be required; and in this we heartily agree with him.—*Medical Record.*

---

*Chloralated Tincture of Iodine.*—An Italian physician employs as a substitute for the ordinary tincture of iodine, to inject into the cavities of the body and in goitre and other tumors, a formula composed of two parts of pure iodine, three parts of chloral hydrate, and fourteen parts of alcohol strength thirty-six. Mix and filter. "The liquid is of a pure golden hue and has an odor and taste which indicate its ingredients." The chloral is dissolved in the solution without any decomposition. The solution is highly commended as a hemostatic and as an antiseptic and hypnotic in large wounds.—*Pacific Medical and Surgical Journal.*

